# Identification of a *Drosophila* Glucose Receptor Using Ca^2+^ Imaging of Single Chemosensory Neurons

**DOI:** 10.1371/journal.pone.0056304

**Published:** 2013-02-13

**Authors:** Tetsuya Miyamoto, Yan Chen, Jesse Slone, Hubert Amrein

**Affiliations:** Department of Molecular and Cellular Medicine, College of Medicine, Texas A&M Health Science Center, College Station, Texas, United States of America; German Institute for Human Nutrition, Germany

## Abstract

Evaluation of food compounds by chemosensory cells is essential for animals to make appropriate feeding decisions. In the fruit fly *Drosophila melanogaster*, structurally diverse chemicals are detected by multimeric receptors composed of members of a large family of Gustatory receptor (Gr) proteins. Putative sugar and bitter receptors are expressed in distinct subsets of Gustatory Receptor Neurons (GRN) of taste sensilla, thereby assigning distinct taste qualities to sugars and bitter tasting compounds, respectively. Here we report a Ca^2+^ imaging method that allows association of ligand-mediated responses to a single GRN. We find that different sweet neurons exhibit distinct response profiles when stimulated with various sugars, and likewise, different bitter neurons exhibit distinct response profiles when stimulated with a set of bitter chemicals. These observations suggest that individual neurons within a taste modality are represented by distinct repertoires of sweet and bitter taste receptors, respectively. Furthermore, we employed this novel method to identify glucose as the primary ligand for the sugar receptor Gr61a, which is not only expressed in sweet sensing neurons of classical chemosensory sensilla, but also in two supersensitive neurons of atypical taste sensilla. Thus, single cell Ca^2+^ imaging can be employed as a powerful tool to identify ligands for orphan Gr proteins.

## Introduction

Taste is a sensory modality found in virtually all animals. Chemicals are detected by specialized sensory cells in the tongue of vertebrates and labial palps and legs of insects, respectively [1]. The main functional sensory units in adult *Drosophila* are the taste sensilla (functionally comparable to mammalian taste buds), which contain two or four Gustatory Receptor Neurons (GRNs) and a single mechanosensory neuron [2], [3] and are distributed among several appendages (labial palp, legs and wings). Electrophysiological recordings from taste sensilla have revealed that the four neurons respond to structurally distinct chemicals [4]. The “sweet” neuron is tuned to sugar compounds, the “bitter/high salt” neuron responds to solutions containing high concentration of salt (>400 mM) and a diverse group of bitter tasting chemicals, the “low salt” neuron is activated by solutions containing low concentration of salt (<200 mM) and the “water” neuron is stimulated by solutions of low osmolarity [5]. The molecular basis for several of these taste modalities is known: sweet and bitter compounds are detected by Gustatory receptor (Gr) proteins which are thought to form multimeric complexes that specifically interact with sugars and diverse organic chemicals (alkaloids, terpenoids etc), respectively [6], [7], [8], [9], [10], [11], while water and salt sensing is mediated by members of the Degenerin/epithelial sodium channel family (Deg/ENaC) of proteins [12], [13], [14]. Sugars, and especially bitter tasting compounds, are structurally diverse, and hence the number of receptors detecting these chemicals is predictably large. Of the 68 Gr proteins in *Drosophila melanogaster*, as many as eight might be expressed in sweet neurons and form multimeric complexes for the detection of sugars [8], [9], [10], [11], while most of the remaining 60 Grs are partially co-expressed in various combinations in bitter/high salt sensing neurons [6], [15], [16] and detect a vast array of non-nutritious chemicals that flies generally avoid [6]. At present, only a handful of Gr proteins have been directly associated with specific chemical ligands. For example, Gr5a is required for trehalose sensing [17], [18], and some but not all of the receptors encoded by the *Gr64* subfamily are necessary for the detection of glucose, sucrose and other sugars [8], [9], [10], [11]. Similarly, Gr66a and Gr93a were shown to be necessary for sensing caffeine, while Gr33a is required for detecting a wide range of bitter tasting chemicals that also include lobeline, quinine and denatonium [7], [19], [20].

Identification of ligands for most of these different Gr proteins was achieved through a combination of electrophysiology and behavioral genetic analyses. However, interpretation of electrophysiological recordings can be ambiguous. Specifically, the spike properties of neurons within a sensillum, the main criteria for assigning activity to a specific neuron type, are often similar and difficult to trace to a particular neuron [21]. Likewise, behavioral analyses of wild type and *Gr* mutant flies can provide direct functional relevance for a receptor's role in sensing a specific compound, but here, data interpretation can be complex due to differences in expression profiles between receptors within a taste modality and functional redundancy between some Grs [7], [9], [10], [11].

Ca^2+^ imaging has become a powerful tool in *Drosophila* neurobiology for the analysis of neural activity. In chemosensation, it is mostly employed to visualize the activity of functionally related neurons (i.e. neurons expressing the same receptor) in their primary processing centers, the antennal lobes or the subesophageal ganglion (SOG), respectively [22], [23], [24], [25]. Here, we present a method whereby neural activity of single taste neurons, associated with taste sensilla on the fifth tarsal segment, is visualized using a Ca^2+^ sensitive fluorescent reporter, GCaMP3.0 [26], expressed under the control of the *Gr61a* and *Gr33a* promoters, respectively (*Gr61a-Gal4* driver and *Gr33a^GAL4^* allele). We show that neurons expressing the bitter receptor gene *Gr33a* are activated by bitter-tasting chemicals such as caffeine, lobeline, quinine and denatonium, as well as high salt (500 mM) solutions, but not by sugars. Likewise, neurons that express *Gr61a-Gal4* respond to carbohydrates, but not to bitter compounds. We find that sugar and bitter response profiles are distinct between neurons and dependent on the sensilla type. Intriguingly, the newly identified, tarsal taste sensilla (5V1) contain a supersensitive sugar neuron that elicits Ca^2+^ responses to sugars at concentration as low as 1 mM. By measuring intracellular Ca^2+^ changes within neurons of flies carrying mutations for these *Gr* genes, we confirm previous electrophysiological recordings that established Gr33a as a receptor component necessary for the detection of many bitter compounds, while also identifying a function for Gr61a as an essential subunit of a glucose receptor. Furthermore, the demonstration that different sweet neurons – as well as different bitter/high salt neurons - show distinct ligand response profiles is consistent with numerous electrophysiological studies [6], [11], [21], [27] and in support of the notion that differential expression of Gr proteins within the same taste modality is the rule, rather than the exception. Thus, our method establishes a reliable and effective alternative to electrophysiological recordings for the characterization of ligand response profiles of taste neurons and the identification of new ligands for orphan Gr proteins.

## Materials and Methods

### Tissue Preparation

**Figure 1:**
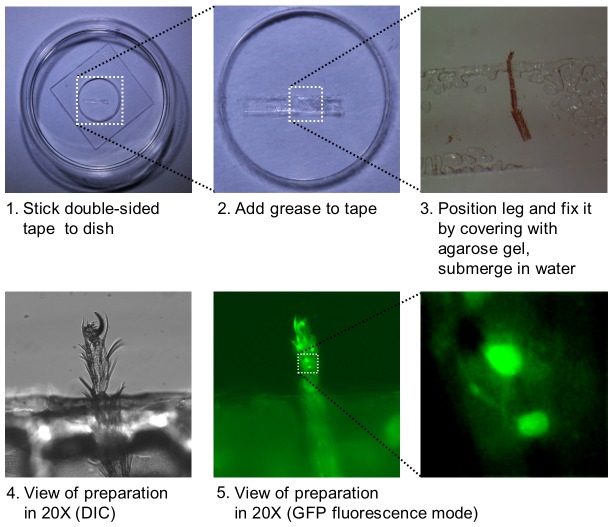
Preparation of forelegs for Ca^2+^ imaging of tarsal GRNs. A double-sided scotch tape is stuck to a 35 mm glass bottom dish (1). A drop of silicon oil is applied on top of the tape (2) and the foreleg, cut between the femur and the tibia, is fixed to the tape, such that the tibia and the upper tarsal segments are covered with oil, while the 4^th^ and part of the 5^th^ tarsal segment are exposed (3). (4) shows a DIC view of the preparation using a 20× objective and the same preparation is shown in (5) and further magnified in using live GFP fluorescence. The two taste neurons expressing G-CaMP3.0 under the control of *Gr61a-GAL4* can be seen.

To prepare tarsi for Ca^2+^ imaging, the foreleg of flies from appropriate genotypes was cut between the femur and the tibia. The tibia and the first three tarsal segments were dipped in silicone oil and placed laterally on double-sided scotch tape that was stuck to a glass bottom dish (Figure 1; MatTek Corp). The tibia and the first three tarsal segments of the leg were covered with 1% agarose, so that only the fourth and fifth tarsal segments were exposed. The whole preparation was then covered with 100 µl of water and immediately used for imaging with a Nikon eclipse Ti inverted microscope.

### Imaging

Imaging was initiated by adding 100 µl of test solutions (2× of the final concentration) by pipette to the preparation, which is submerged in 100 µl of water. Images were acquired every 500 ms, 20 frames before application (10 s) and 60 frames after application (30 s) of ligand. Each preparation was tested with 2–4 different compounds. Imaging was performed with a Nikon 20× water objective and a Lumen 200 light source (Prior Scientific Inc). Samples were excited at 488 nm (metal halide lamp), and emitted light was collected through a 515–555 nm filter. Data acquisition was performed with NIS-Elements software (Nikon). To calculate max ΔF/F %, measurements were taken in the cell bodies or at the base of axons. Adjacent regions were used to determine background auto fluorescence. Average of five frames taken immediately before the application of ligand was defined as a baseline. Max ΔF/F % represented the highest value within 30 seconds after ligand application. For most but the lowest concentrations tested, max ΔF/F was reached between 1 and 3 seconds after ligand application, whereas for the lowest concentrations tested, max ΔF/F was reached between 20 and 30 seconds after application.

### Genetics

Expression analysis was carried out on *w^1118^*; *Gr33a^GAL4^/UAS-mCD8RFP*; *Gr64f^LexA^ lexAop-rCD2GFP* using live imaging on a Nikon A1 confocal microscope. Ca^2+^ imaging data of bitter/high salt neurons were obtained from flies of the following genotypes: *Gr33a^GAL4^/UAS-GCaMP3.0* (wild type control) and *Gr33a^GAL4^/Gr33a^1^ UAS-GCaMP3.0* (*Gr33a* mutant). Both *Gr33a^GAL4^* and *Gr33a^1^* were generated by homologous recombination [19]. Ca^2+^ imaging data of sweet neurons were obtained from flies of the following genotypes: *UAS-GCaMP3.0/Gr61a-Gal4*; (wild type control), *UAS-GCaMP3.0/Gr61a-Gal4; ΔGr61a/ΔGr61a* (*Gr61a* mutant) and *UAS-GCaMP3.0 UAS-Gr61a/Gr61a-Gal4*; *ΔGr61a/ΔGr61a* (*Gr61a* rescue). *ΔGr61a* is a deletion mutation [11]. For PER, the following genotypes were tested: *w^1118^* (wild type), *ΔGr61a/ΔGr61a* (mutant), *Gr61a-Gal4*; *ΔGr61a/ΔGr61a* (mutant/control), *UAS-Gr61a*; *ΔGr61a/ΔGr61a* (mutant/control) and *UAS-Gr61a/Gr61a-Gal4*; *ΔGr61a/ΔGr61a* (rescue).

### Proboscis Extension Reflex (PER) assay and statistical analysis

PER assays were essentially carried out as described by Slone et al [9], with minor modifications. Briefly, flies were collected within 12 hrs of eclosion and kept on standard food for 2 to 5 days. The flies were starved for 25 to 30 hrs in vials with a water-saturated Whatman paper. Flies were immobilized on ice, rather than carbon dioxide, and mounted on their backs on a microscope slide using double-sided scotch tape. After mounting, flies were allowed to recover for ∼2 hours, and prior to testing their response to sugar solutions, they were allowed to drink water *ad libitum*. Flies not responding to water were excluded. A PER was recorded if a fly extended the proboscis after a tastant was applied to the forelegs. Each fly was tested with each sugar once, and flies were allowed to drink water between each application. Five flies were tested in any given experiment. Error bars represent the standard error of the mean (SEM), and statistical significance was calculated using ANOVA.

### Chemicals

All sugars (crystalline (D) form), salts, base and acid were purchased from Sigma-Aldrich, with purity >99%. Caffeine (Sigma-Aldrich #C53) was of >99% purity, while quinine hydrochloride (Sigma-Aldrich #Q1125), denatonium benzoate (Sigma-Aldrich #D5765) and lobeline hydrochloride (Tokyo Chemical Industry Co. LTD #L0096) were of >98% purity.

## Results

### Chemosensory sensilla on the tarsi

**Figure 2:**
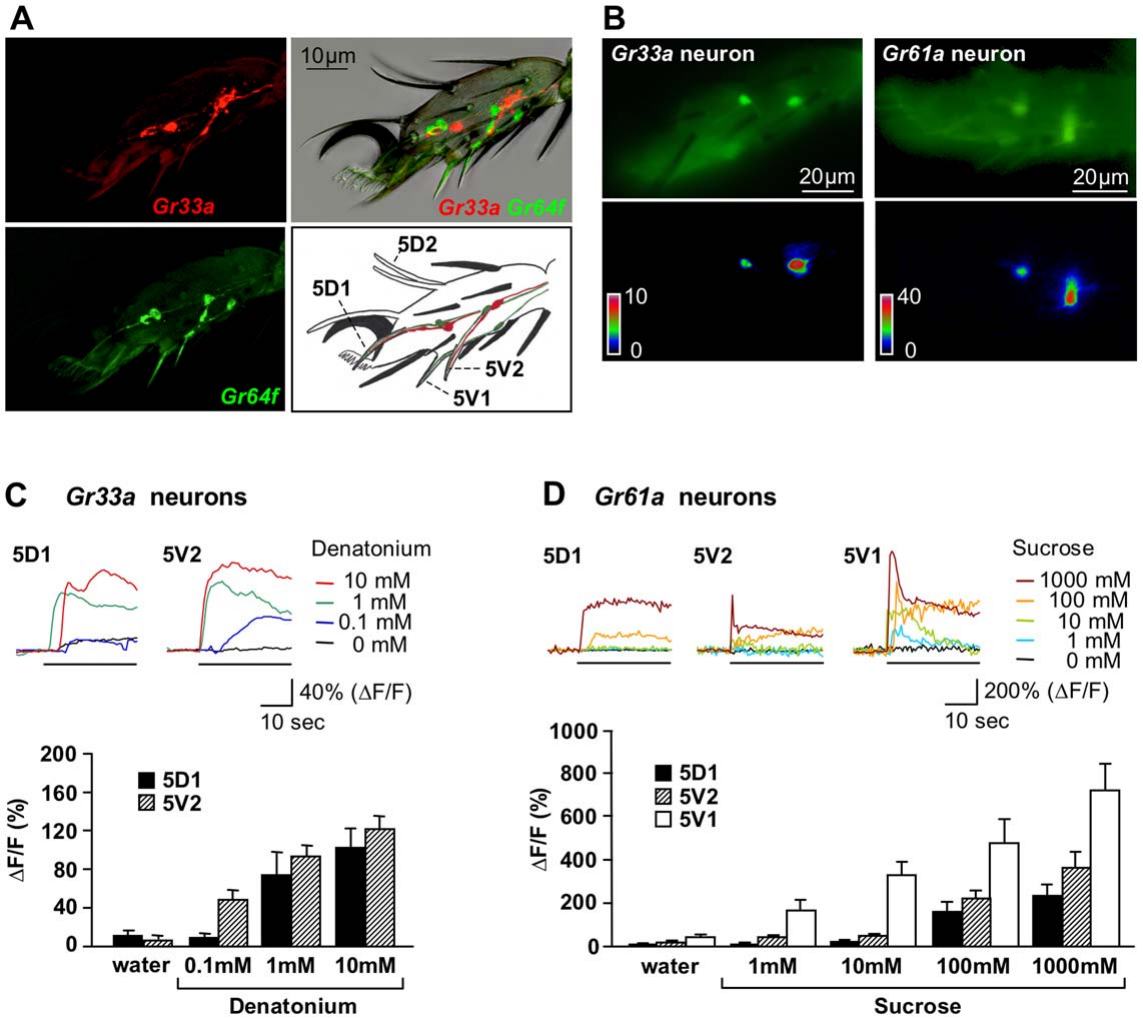
Concentration-dependent Ca^2+^ responses of bitter and sweet neurons. (A) Expression of *Gr33a^GAL4^* and *Gr64f^LexA^* in GRNs of the fifth tarsal segment. *Gr64f^LexA^* is completely co-expressed with *Gr61a-GAL4* (JS and HA, unpublished). The image at the top right shows live expression of mCD8RFP and rCD2GFP in bitter/high salt (red) and sweet neurons (green), respectively, laid over the phase-contrast image. Identification of neural processes is possible in the images where the two fluorescent markers are visualized separately. Note that the 5V1 sensilla contain only a *Gr64f*, but not a *Gr33a-* expressing neuron. The drawing identifies each of the chemosensory sensilla. Also, only one chemosensory bristle/neuron of each pair is visible from a side view, with the exception of 5D2, which harbors neither a *Gr33a^GAL4^* nor a *Gr64f^LexA^ Gr* expressing neuron. The single, long sensilla at the tip (above the claw) has a morphology typical of chemosensory bristles, but neither of the drivers is expressed in its associated neurons. Mechanosensory bristles are shown in black. (B) Images of bitter/high salt (*Gr33a*) and sweet neurons (*Gr61a*) expressing *UAS-GCaMP3.0* in the 5^th^ tarsal segment of forelegs. The upper panels show tarsal neurons labeled *with Gr33a^GAL4^/UAS-GCaMP3.0* (left) and *Gr61a-GAL4/UAS-GCaMP3.0* (right), respectively, before application of ligand. The lower panel shows the increase of fluorescence (ΔF) coded as pseudocolor images focused on one of the neurons of each leg after application of 1 mM denatonium and 100 mM sucrose, respectively. (C) Dosage dependent intracellular calcium changes (%ΔF/F) of representative samples in 5D1- and 5V2- associated bitter/high salt neurons (top graph). The black line indicates stimulus application. Average of maximum responses for the pair 5D1- associated neurons were similar and pooled, as were the responses for the pair 5V2- associated neurons (bottom graph). Genotype: *Gr33a^GAL4^/UAS-GCaMP3.0.* 3<n<12; ANOVA: * p<0.05, ** p<0.001. (D) Dosage dependent intracellular calcium changes (%ΔF/F) of representative samples in the 5D1-, 5V1- and 5V2- associated sweet neurons (top graph). The black line indicates stimulus application. Average of maximum responses for neurons of a given pair were similar and therefore pooled (bottom graph). Genotype: *Gr61a-GAL4/UAS-GCaMP3.0.* 7<n<12; ANOVA: * p<0.05, ** p<0.001.

The fifth segment of the *Drosophila* leg features at least four pairs of chemosensory sensilla, which we named based on their segmental, dorso-ventral and anterior-posterior location (1 to 5 for segment, D/V for dorsal/ventral and 1 to n, from anterior to posterior; Figure 2A, Table S1). Three of these sensilla, 5D1, 5D2 and 5V2, were previously described by Meunier and co-workers [21], [28], [29]. The fourth sensillum, 5V1, features a short and straight bristle and is described and characterized here for the first time. The 5D1 and 5V2 sensilla have been characterized mainly for their response to bitter chemicals using single sensilla recordings [21], while the response properties of 5V1- and 5D2- associated neurons have not yet been investigated. These four sensilla are present as symmetrical pairs (one located on the medial and one on the lateral side of the leg), and our Ca^2+^ imaging studies and previous single sensilla recordings have found no differences between neurons of a given pair. Therefore, throughout this paper, no distinction is made between measurements of a given pair, and the respective data are pooled.

We first mapped expression of *Gr33a* and *Gr61a* using *Gr33a^GAL4^* and *Gr64f^LexA^* knock-in alleles (Figure 2A), the latter being precisely co-expressed with *Gr61a-GAL4* (JS and HA, unpublished data) [11], [19]. *Gr64f^LexA^* is expressed in a single neuron of three of the four sensilla pairs (5D1, 5V1 and 5V2), while *Gr33a^Gal4^* is expressed in another neuron of the 5D1 and 5V2 sensilla, but not the 5V1 sensilla. Neither of the drivers is expressed in neurons of the 5D2 sensilla, nor the single chemosensory-like sensillum at the tip of the 5^th^ segment, featuring a long, curved bristle (Figure 2A). Hence, no Ca^2+^ imaging data could be obtained from neurons associated with these sensilla.

### *Ex vivo* preparation of tarsal taste neurons

To facilitate recording of ligand mediated neural activity of single taste neurons, we employed a Ca^2+^ imaging assay using the foreleg (for details, see Material and Methods). This preparation consists of the tibia and all five tarsal segments of the foreleg: the three most proximal tarsal segments along with the tibia are embedded in agarose, while the fourth and fifth tarsal segments are protruding into the dish, where they can be exposed to the test solution (Figure 1; for details, see Material and Methods). The preparation is equilibrated in water, before it is challenged with chemicals. While the work presented here is confined to 5D1, 5V1 and 5V2 sensilla, the Ca^2+^ imaging method can be performed on any sensilla located on the two most distal segments, the only limitation being that a *Gal4* driver is available.

We first tested our preparation by measuring responses to denatonium and sucrose, two ubiquitous ligands known to activate bitter and sweet neurons, respectively. Denatonium elicited dosage dependent Ca^2+^ responses in all four neurons expressing the calcium indicator GCaMP3.0 under the control of *Gr33a^GAL4^* (Figures 2B and 2C), which is consistent with previous electrophysiological recordings from tarsal sensilla [21]. Likewise, sucrose elicited Ca^2+^ responses in all six putative sweet neurons in which GCaMP3.0 is expressed under the control of the *Gr61a-GAL4* driver (Figure 2B and 2D). Remarkably, the sweet neurons associated with the atypical taste sensilla (5V1) showed much stronger responses than the neurons associated with either the 5D1 or the 5V2 sensilla (Figure 2D). Responses to both denatonium and sucrose occurred at physiologically relevant concentrations, established both in behavioral analyses and electrophysiological recordings [6], [11], [21], [27]. Taken together, these experiments show that the tarsal preparation can efficiently be used to assess physiological responses from individual tarsal taste neurons.

### Distinct subtypes of both sweet and bitter/high salt neurons

**Figure 3:**
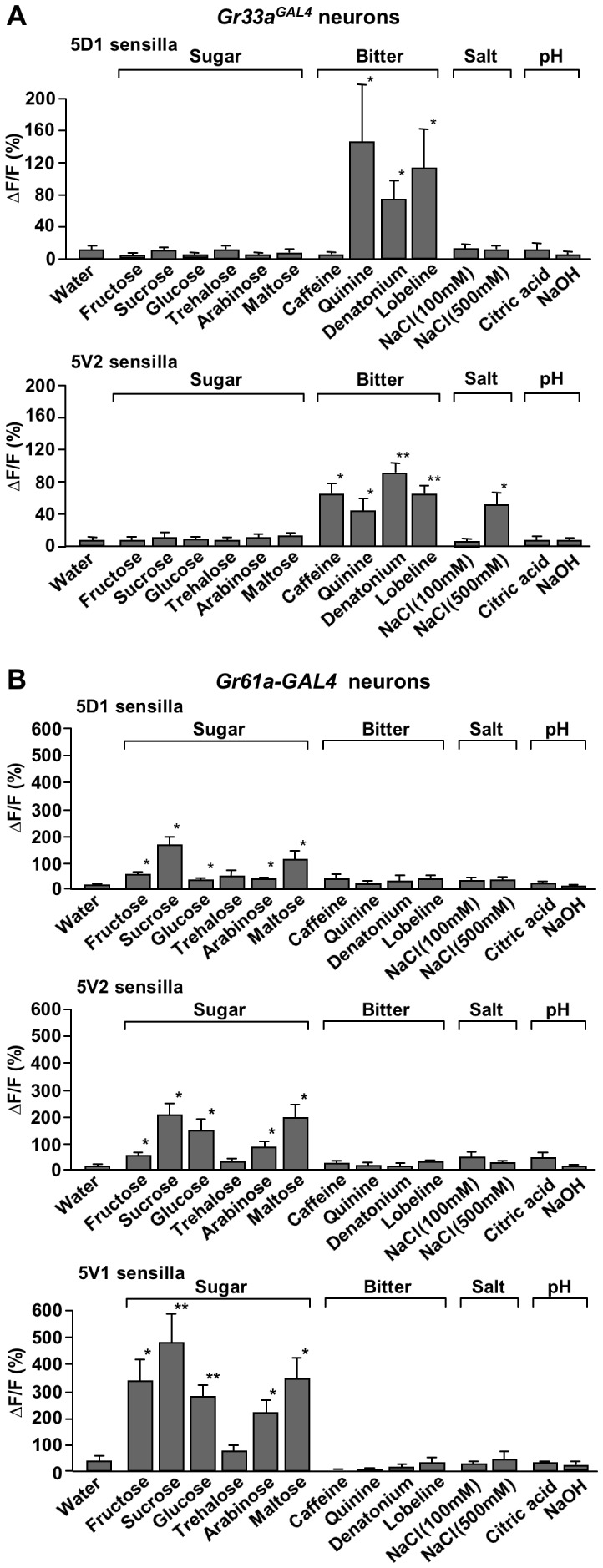
Subtypes of neurons within a taste modality show different response profiles. Ca^2+^ responses of *Gr33a^GAL4^* (A) and *Gr61a-GAL4* expressing neurons, stimulated by various sugars, bitter compounds, low (100 mM) and high (500 mM) NaCl, acidic (citric acid, pH 2.5) and basic (NaOH, pH 12) solutions. Concentrations were 100 mM for sugars, 10 mM for caffeine and 1 mM for quinine, denatonium and lobeline. (A) All *Gr33a^GAL4^* neurons respond to all bitter compounds tested. Note that the intensity of the response is different in the two types of neurons: The 5D1- associated neurons respond best to quinine, followed by lobeline and denatonium, but do not respond to caffeine and high salt, while the 5V2- associated neurons respond best to denatonium, followed by caffeine and lobeline, high salt and quinine. Note that six 5V2- associated neurons barely responded to quinine (5.2+/−1.3), while eleven responded robustly (ΔF/F = 59.6+/−11.3). Neither the 5D1- nor the 5V2- associated bitter/high neurons respond to sugars, high and low pH or low salt. 3<n<17 for bitter compounds; 4<n<8 for all other compounds. ANOVA: * p<0.05, ** p<0.001. (B) All *Gr61a-GAL4* expressing sweet neurons respond to sugars, but not to other chemicals. Absolute response is largest in 5V1- associated neurons, followed by 5V2- and 5D1- associated neurons. Also note that the relative intensity to various sugars is different in the three sweet neurons (for details, see text). 7<n<12 for sugars; 3<n<7 for all other compounds. ANOVA: * p<0.05, ** p<0.001.

To establish ligand response profiles of sweet and bitter/high salt neurons, we carried out Ca^2+^ imaging experiments with numerous, chemically diverse organic compounds, as well as salts and acids (Figure 3): six sugars (fructose, sucrose, glucose, trehalose arabinose and maltose), four bitter compounds (caffeine, quinine, denatonium and lobeline), two concentrations of NaCl (100 and 500 mM), citric acid and NaOH. We first evaluated the response of *Gr33a^GAL4^* expressing bitter/high salt neurons associated with the 5D1 and 5V2 sensilla (Figure 3A). The *Gr33a^GAL4^* expressing neurons showed robust responses to the three bitter compounds quinine, denatonium and lobeline, and the 5V2-, but not the 5D1- associated neurons, were also activated by caffeine and 500 mM NaCl. Moreover, the 5V2– associated Ca^2+^ responses to quinine were significantly smaller than those of 5D1- associated neurons, in part because about 1/3 of the neurons exhibited negligible responses to this compound (ΔF/F = 5.2+/−1.3, n = 6), while the remainder 2/3 responded robustly (ΔF/F = 59.6+/−11.3 n = 11). Finally, as expected, none of the sugars, citric acid or NaOH activated any of the *Gr33a^GAL4^* expressing neurons.

When we challenged *Gr61a-Gal4* expressing neurons with the same panel of chemicals, we observed responses to sugars only (Figure 3B). We note that the 5V1- associated neurons which exhibited higher responses to sucrose than all other neurons (Figure 2D), produced also higher Ca^2+^ increases when stimulated with other sugars (Figure 3B). Moreover, notable differences in the Ca^2+^ response profile between the three types of sweet neurons were apparent. In 5V2- associated neurons, the response was highest to sucrose, followed by maltose, fructose/trehalose, and glucose/arabinose. In 5D1– associated neurons, the order was sucrose/maltose, glucose, arabinose, fructose and trehalose, while in 5V1 - associated neurons, it was sucrose, followed by fructose/maltose, glucose, arabinose and trehalose. Taken together, these experiments are consistent with electrophysiological recordings, which hold that the two modalities of bitter and sweet taste are mediated by distinct group of neurons. In addition, they confirm and further extend observations suggesting that different subtypes of neurons exist within either the bitter/high salt or sweet taste modality [6], [11], [21], [27], likely a consequence of differences in *Gr* gene expression profiles between neurons.

### Ca^2+^ imaging in mutant flies: identification of ligands for Gr proteins

**Figure 4:**
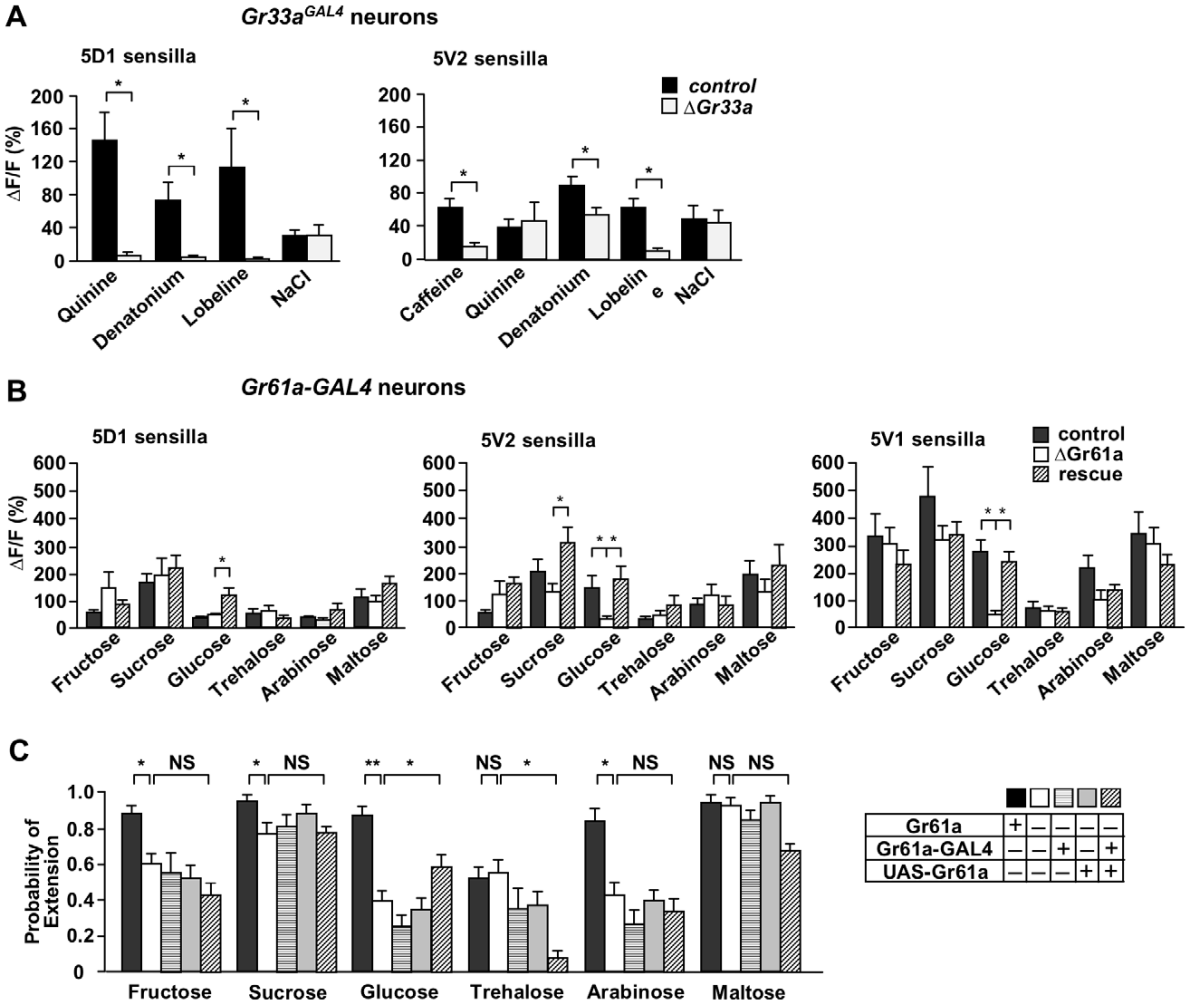
*Gr33a* and *Gr61a* are necessary for sensing bitter compounds and glucose, respectively. (A) *Gr33a* is necessary for sensing all bitter compounds in 5D1- associated bitter/high salt neurons, but not in 5V2- associated neurons. In the latter, response to caffeine and lobeline are largely eliminated in homozygous *Gr33a^GAL4^* mutants, while response to denatonium and quinine are either reduced or unaffected, respectively. Response to high salt was not affected. Concentrations were 10 mM for caffeine, 1 mM for quinine, denatonium and lobeline and 500 mM for NaCl. Genotypes: *Gr33a^GAL4^/UAS-GCaMP3.0* (control) and *Gr33a^GAL4^/Gr33a^1^ UAS-GCaMP3.0* (*ΔGr33a*). 3<n<17 for bitter compounds; 4<n<8 for NaCl. ANOVA: * p<0.05, ** p<0.001. (B) *Gr61a* is essential for sensing glucose, but not other sugars. Only response to glucose is eliminated in 5V1- and 5V2- associated sweet neurons (note that the 5D1- associated neurons show only a very small response to glucose). While the response to some of the other sugars is slightly reduced (i.e. sucrose and arabinose) in the 5V1- and 5V2- associated neurons of *Gr61a* mutants when compared to controls, this difference is statistically not significant. Concentrations were 100 mM for all sugars. Genotypes: *UAS-GCaMP3.0/Gr61a-Gal4*; (control), *UAS-GCaMP3.0/Gr61a-Gal4; ΔGr61a/ΔGr61a* (*ΔGr61a*) and *UAS-GCaMP3.0 UAS-Gr61a/Gr61a-Gal4; ΔGr61a/ΔGr61a* (rescue). 7<n<12. ANOVA: * p<0.05, ** p<0.001. (C) PER response to the sugar glucose is significantly reduced in *Gr61a* mutant flies, but partially rescued by expressing a *UAS-Gr61a* transgene. Overall reduced PER response to sugars in *Gr61a* mutants is not due to the lack of the *Gr61a* gene, since PER does not increase in the rescue flies. Genotypes: wild type: *w^1118^* (black box), mutants: *ΔGr61a/ΔGr61a* (white box), *Gr61a-Gal4*; *ΔGr61a/ΔGr61a* (horizontal crosshatched), *UAS-Gr61a*; *ΔGr61a/ΔGr61a* (gray), and rescue: *UAS-Gr61a/Gr61a-Gal4*; *ΔGr61a/ΔGr61a* (diagonal crosshatched). A single experiment was the result of three to five applications. 11<n<20, ANOVA. P<0.05.

We next sought to demonstrate that single cell Ca^2+^ imaging can be utilized to identify ligands for Gr proteins. We first asked whether Ca^2+^ responses to specific ligands were reduced or abolished in *Gr33a-*expressing bitter neurons of flies with mutations in *Gr33a*. Previously, *Gr33a* was shown to be necessary for sensing many bitter compounds [19]. When neurons of *Gr33a* mutant flies were imaged, we found that Ca^2+^ responses of 5D1- associated bitter neurons were completely lost to all tested bitter compounds, while those of 5V2- associated bitter neurons were reduced for caffeine, denatonium and lobeline, but not quinine (Figure 4A). The loss of function phenotype in 5D1– associated neurons is consistent with electrophysiological recordings from labellar taste sensilla, which indicated that Gr33a is a major component of a receptor with broad specificity to many bitter tasting chemicals [19]. However, 5V2– associated neurons do not require Gr33a for sensing at least some bitter compounds (such as quinine). Thus, while Gr33a clearly contributes to bitter sensing, additional receptors must be co-expressed in 5V2- associated neurons that can partially compensate for the loss of Gr33a.

Next, we assessed the effects of the *ΔGr61a* mutation on the cellular response to various sugars (Figure 4B). Previous electrophysiological analyses did not reveal any significant phenotype to a broad range of sugars, including the ones tested here [11]. When we compared *Gr61a*-expressing sweet neurons of *Gr61a^+^* and *ΔGr61a* flies, no significant differences in Ca^2+^ responses were observed when stimulated with fructose, sucrose, trehalose, arabinose or maltose. However, 5V2- and 5V1- associated sweet neurons of *ΔGr61a* flies showed virtually no response to glucose, while this sugar elicited a robust response in corresponding neurons of *Gr61a^+^* control flies. Importantly, when *ΔGr61a* flies were complemented with a *UAS-Gr61a* transgene driven by *Gr61a-GAL4*, complete restoration of the Ca^2+^ response to glucose was observed. Interestingly, albeit the 5D1- associated neurons of both control and *ΔGr61a* flies show only a negligible response to glucose, these neurons exhibited a significant increase in the response to this sugar when expressing the *UAS-Gr61a* transgene. Similarly, we observed an increase in the response to sucrose (which contains a glucose moiety) in 5V2- associated sweet neurons expressing the *UAS-Gr61a* transgene, compared to homozygous mutants and controls. We suggest that overexpression of Gr61a in these neurons increases protein levels of a functional glucose/sucrose receptors, thereby increasing the sensitivity of the neurons to these sugars. Regardless, our Ca^2+^ imaging studies clearly show that Gr61a is an integral component of a glucose receptor in some tarsal chemosensory sensilla.

Lastly, we investigated whether *Gr61a* is also necessary for the behavioral response to glucose. We performed Proboscis Extension Reflex (PER) assays in *w^1118^* (wild type control) flies, *ΔGr61a* homozygous mutant flies with or without either the *UAS-Gr61a* transgene or the *Gr61a-GAL4* driver (mutant/controls) and with both transgenes (rescue). *w^1118^* flies showed higher PER responses than all other flies for several sugars, indicating that the *ΔGr61a* strain exhibits a reduced, non-specific behavioral deficits to sweet tasting chemicals. When the *Gr61a-Gal4* driver and the *UAS-Gr61a* transgene were crossed into *ΔGr61a* homozygous flies, the only significant PER increase was observed with glucose solutions. Thus, our Ca^2+^ imaging and PER analyses establish that Gr61a is necessary for glucose sensing in a subset of sweet neurons. We note that PER response to trehalose decreased in the rescue flies; one possibility for this is that altered Gr stoichiometry caused by Gr61a overexpression increases the amount of one receptor (glucose) at the expense of another (trehalose) in some neurons (see also reduced trehalose response of 5D1- associated sweet neuron in “rescue” flies; Figure 4B).

## Discussion

We have established a Ca^2+^ imaging method for visualizing and recording neural activity of single GRNs. This efficient experimental strategy is well suited to assess ligand-mediated neural responses in wild type and *Gr* mutant flies. While single sensilla recordings can reveal information about the electrical properties of neurons that may not be obtained with Ca^2+^ imaging (spike amplitude/frequency, precise temporal resolution of activity etc), the latter has the distinct advantage of unambiguous cellular resolution. In addition, the use of live GFP markers, a necessary component of Ca^2+^ imaging applications, led to the identification of a morphologically atypical chemosensory sensilla, 5V1, whose sweet neuron is supersensitive. This raises the possibility that additional taste bristles might be “hidden” in the broadly distributed chemosensory system of the fly.

### Correlation of electrophysiological recording and Ca^2+^ imaging

The sensitivity of our Ca^2+^ imaging assay is comparable to that of electrophysiological recordings [21]. For example, the same concentration of bitter chemicals is required to reliably activate bitter/high salt neurons (∼1 mM for denatonium, lobeline and quinine and ∼10 mM for caffeine) (Figure 2 and 3 and [21]). No dose response profiles have been reported for sugars using electrophysiological recordings in tarsal sensilla, but Hiroi and colleagues tested numerous sugars at different concentrations of selected labellar sensilla and found that ∼10 mM concentration (sucrose) is sufficient to generate a noticeable increase in firing frequency [27]. This compares well to the sweet neuron of the 5D1 and 5V2 sensilla. Interestingly, the newly discovered 5V1- associated sweet neuron appears significantly more sensitive than the 5D1- and 5V2- associated sweet neurons (Figure 2).

At this time, it is difficult to compare the two methods with regard to the distinct response profiles of specific sweet or bitter neurons, due to the small overlap in the number of characterized sensilla and ligands. Nevertheless, some notable similarities emerge: Consistent with our imaging data (Figure 3A), Meunier and co-workers [21] found that 5D1- associated sensilla strongly responds to quinine but not to caffeine, while the 5V2- associated sensilla responded to berberine and caffeine, but not to (low concentrations of) quinine. However, the 5V2- associated sensilla did respond to 10 mM quinine with “erratic bursts of action potentials”. This observation is reminiscent of our result, which revealed that approximately one third of 5V2- associated bitter/high salt neurons showed little or no response to quinine, while the other 2/3 were readily activated by this ligand (see above). We also note that a comprehensive electrophysiological characterization found distinct response profiles of individual labellar taste sensilla to bitter chemicals [6], an observation consistent with our studies. These differences are likely brought about by distinct *Gr* expression profiles in different bitter/high salt neurons [6], [15], [16].

Previous electrophysiological and behavioral analyses showed that *Gr33a* is essential for sensing quinine, denatonium, lobeline and caffeine [7], and the authors of that study suggested that Gr33a may be a common subunit in receptors for sensing a diverse range of bitter chemicals. A similar conclusion may be drawn from Ca^2+^ imaging of the 5D1- associated neurons (Figure 4). However, responses of the 5V2- associated bitter/high salt neuron indicate that while Gr33a is an important receptor component for detecting many bitter compounds, it is not absolutely required to sense quinine and denatonium in these cells. We suggest that another Gr present in these cells can compensate for the absence of Gr33a, or alternatively, that these chemicals are detected by yet another set of receptors, such as members of the ionotropic glutamate receptor family, many of which are expressed in the gustatory system [30].

### Gr61a is a glucose receptor

Gr61a, a member of the putative sugar receptor subfamily, is broadly co-expressed in sweet cells with Gr5a and Gr64f, which are required for sensing trehalose and many other sugars, respectively [11]. Moreover, the *Gr61a* gene is conserved throughout the *Drosophila* lineage [31], [32]. Surprisingly, electrophysiological analyses of labellar taste sensilla in wild type and *ΔGr61a* mutant flies did not reveal a function for this gene in sugar sensing [11]. However, we find that tarsal sweet sensing taste neurons exhibit a dramatic decrease in glucose sensing in 5V2- and 5V1- sensilla of *ΔGr61a* flies, compared to control and rescue flies (Figure 4B). Although PER to several sugars was lower in *ΔGr61a* flies than controls (probably due to genetic modifiers in this strain), only the response to glucose significantly increased in the presence of a *Gr61a* transgene (Figure 4C), indicating that Gr61a is necessary for both cellular and behavioral responses to this sugar. The residual PER response to glucose in *ΔGr61a* flies (Figure 4), as well as the electrophysiological response to glucose of L-type sensilla in the labellum of such flies [11], argues for functional redundancy between putative sugar receptors. For example, an additional *psGr* gene might be functionally redundant and co-expressed with *Gr61a* in labellar taste sensilla, which would explain the lack of a glucose sensing phenotype in labellar sweet neurons.

Based on electrophysiological recordings from L - type labellar taste sensilla and behavioral studies using flies containing partial *Gr64* gene deletions, it was suggested that sugar sensing is mediated by only three of the eight putative sugar receptor genes: *Gr5a*, *Gr64a* and *Gr64f* [10], [11]. Based on our findings presented here, it is apparent that the detection of glucose involves at least one additional members of this subfamily, Gr61a. Finally, we note that flies lacking all eight putative sugar receptor still respond to fructose and sucrose, which is mediated by yet another Gr protein, Gr43a [33].

## Supporting Information

Table S1 **Identity of sensilla and their bitter/sweet neurons expressing the two** ***GAL4*** **drivers used in this study,** ***Gr61a-GAL4*** **and** ***Gr33a^GAL4^*** **.**
(DOCX)
