# Supplementary material for: Identification of a Drosophila Glucose Receptor Using Ca2+ Imaging of Single Chemosensory Neurons
Source: PLoS One. 2013 Feb 13;8(2):e56304. doi: 10.1371/journal.pone.0056304 (PMC3571953; doi:10.1371/journal.pone.0056304)
Supplement: Table S1 — Identity of sensilla and their bitter/sweet neurons expressing the two GAL4 drivers used in this study, Gr61a-GAL4 and Gr33aGAL4 . (DOCX) [file pone.0056304.s001.docx]

**TABLE S1:** **Identity of sensilla and their bitter/sweet neurons expressing the two *GAL4* drivers used in this study, *Gr61a-GAL4* and *Gr33a^GAL4^*.**

| \| This study \| 5D1 \| 5D2 \| 5V1 \| 5V2 \| \| --- \| --- \| --- \| --- \| --- \| \| Meunier et al. (2003) \| 5b \| 5a \| - \| 5s \| \| *Gr61a/Gr33a* expressing neurons \| Yes/yes \| No/no \| Yes/no \| Yes/yes \| |
| --- | --- | --- | --- | --- | --- | --- | --- | --- | --- | --- | --- | --- | --- | --- | --- |

The top row identifies the four pairs of chemosensory sensilla as referred in this study based on their segmental (5) and dorsal vs ventral (D/V) location. The last number indicates the anterior (low) to posterior position of the sensilla within the segment. The second row indicates the nomenclature used by Meunier et al., (2002 and 2003). The third row indicates whether or not the sensilla harbors a *Gr61a-GAL4* and a *Gr33a^GAL4^* expressing neurons. Note that all sensilla appear as pairs, one on the medial and one on the lateral side of the leg.

**REFERENCES:**

Meunier N, Marion-Poll F, Rospars JP, Tanimura T (2003) Peripheral coding of bitter taste in Drosophila. J Neurobiol 56: 139-152.
